# Variation of *Fusarium* Free, Masked, and Emerging Mycotoxin Metabolites in Maize from Agriculture Regions of South Africa

**DOI:** 10.3390/toxins12030149

**Published:** 2020-02-28

**Authors:** Theodora Ijeoma Ekwomadu, Toluwase Adeseye Dada, Nancy Nleya, Ramokone Gopane, Michael Sulyok, Mulunda Mwanza

**Affiliations:** 1 Department of Biological Sciences, Faculty of Natural and Agricultural Sciences, Mafikeng Campus, North-West University, Mmabatho 2735, South Africa; ramokone.gopane@gmail.com; 2 Department of Animal Health, Faculty of Natural and Agricultural Sciences, Mafikeng Campus, North-West University, Mmabatho 2735, South Africa; adedad@gmail.com (T.A.D.); dangwa@gmail.com (N.N.); mulunda.mwanza@nwu.ac.za (M.M.); 3 Department of Agro Biotechnology (IFA-Tulln), University of Natural Resources and Life Sciences Vienna (BOKU), A-3430 Tulln, Austria; michael.sulyok@boku.ac.at

**Keywords:** *Fusarium*, maize, masked mycotoxins, LC-MS/MS, agricultural regions, South Africa

## Abstract

The presence of mycotoxins in cereal grain is a very important food safety issue with the occurrence of masked mycotoxins extensively investigated in recent years. This study investigated the variation of different *Fusarium* metabolites (including the related regulated, masked, and emerging mycotoxin) in maize from various agriculture regions of South Africa. The relationship between the maize producing regions, the maize type, as well as the mycotoxins was established. A total of 123 maize samples was analyzed by a LC-MS/MS multi-mycotoxin method. The results revealed that all maize types exhibited a mixture of free, masked, and emerging mycotoxins contamination across the regions with an average of 5 and up to 24 out of 42 investigated *Fusarium* mycotoxins, including 1 to 3 masked forms at the same time. Data obtained show that fumonisin B_1,_ B_2,_ B_3,_ B_4_, and A_1_ were the most prevalent mycotoxins and had maximum contamination levels of 8908, 3383, 990, 1014, and 51.5 µg/kg, respectively. Deoxynivalenol occurred in 50% of the samples with a mean concentration of 152 µg/kg (max 1380 µg/kg). Thirty-three percent of the samples were contaminated with zearalenone at a mean concentration of 13.6 µg/kg (max 146 µg/kg). Of the masked mycotoxins, DON-3-glucoside occurred at a high incidence level of 53%. Among emerging toxins, moniliformin, fusarinolic acid, and beauvericin showed high occurrences at 98%, 98%, and 83%, and had maximum contamination levels of 1130, 3422, and 142 µg/kg, respectively. Significant differences in the contamination pattern were observed between the agricultural regions and maize types.

## 1. Introduction

Mycotoxins can adversely affect human and animal health condition, productivity, economics, and trade [1,2,3]. The United Nations’ Food and Agricultural Organization (FAO) made an estimate that there was significant contamination of about twenty-five percent of the world’s food crops with mycotoxins leading to annual loss in the range of one million tons [4]. Recently, studies suggest that the percentage of contaminated cereals is much higher at 72% [5]. The difference may be due in part, to what levels are considered as contamination, in addition to advances in detection and monitoring [6]. Recently, it has come to be clearer that in mycotoxin-contaminated products, various structurally-related compounds produced during plant metabolism or during after food processing can co-occur with the parent toxins [7]. These mycotoxin derivatives had a very different chemical behavior including polarity and solubility, compared to the precursor and thus, can easily escape routine analyses [8]. Since they are undetectable by conventional analytical techniques because of their altered structures, there is thus generally an underestimation of the mycotoxin load. Also, despite their chemical alteration, coupled with the fact that they are generally not regulated by legislation, they may be considered as being masked [6]. Furthermore, it has to be highlighted that masked mycotoxins can be “unmasked” again in the digestive tract of animals and humans, releasing the parent compound with its toxicological effects again. A similar situation exists with emerging mycotoxins: toxicological data are scarce which makes it difficult to set up regulations and maximum tolerated limits to protect humans and animals from potential health risks. On the other hand, “emerging mycotoxins” generally represents a group of chemically altered mycotoxins which have no regulations up till date. Studies employing LC–MS/MS for structure elucidation provide insights about these newly discovered metabolites [9]. Common emerging *Fusarium* mycotoxins are eniantins (ENNs), beauvericin (BEA), moniliformin (MON), fusaproliferin (FP), fusidic acid (FA), culmorin (CUL), and butenolide (BUT). Jestoi [10] published an extensive review regarding this diverse set of chemical compounds.

Conjugated or masked mycotoxins first came to the attention of public health officials, when animals fed with apparently low mycotoxin contaminated feed, showed high severity of mycotoxicosis. The unanticipated high toxicity was ascribed to the presence of undetected, conjugated forms of mycotoxins [8].

Historically, Gareis et al., [11] for the first time, used the term ‘masked mycotoxins’ and it refers to the products that are formed when plants metabolize mycotoxins, as part of their natural defense system. These secondary metabolites are not detectable by conventional techniques because their structure has been altered in the plant, nor are they regulated. The metabolites are so-called masked as they become toxic again as soon as they cleave off their sugar molecule in the intestine of the humans and animals. The term conventional applies to the analytical detection methods that have previously or initially been developed for specific mycotoxins only. Then in 2013, researchers revisited the masked mycotoxin topic again and Berthiller et al. [8] made a clear definition of what masked mycotoxins are. The term “masked mycotoxins” is now widely accepted. However, in 2014, Michael Rychlik and his research group came up with a comprehensive definition to include all modified forms of mycotoxins as well as masked mycotoxins as “modified mycotoxins” [12]. Actually, the latter is the umbrella term of all mycotoxins that are modified by some sort of process (for example, food processing). Masked mycotoxins are sort of, part of this definition, but only entail the molecules that are formed by the plants.

The likelihood of mobilization of mycotoxins that interact with metabolically active plants in the field is the issue. As *Fusarium* infection usually occurs in the field (in contrast to *Aspergillus* and *Penicillium* infections), the *Fusarium* mycotoxins (deoxynivalenol, zearalenone, fumonisins, nivalenol, fusarenon- X, T-2 toxin, HT-2 toxin) are the most prominent target for conjugation [8]. Although, transformation of other mycotoxins e.g., ochratoxin A, patulin, and destruxins, by plants has also been described. Specifically, deoxynivalenol-3-glucoside (D3G), zearalenone-14-glucoside (Z14G), and zearalenone-14-sulphate (Z14S) are the most commonly found masked mycotoxins occurring in food commodities [8]. Currently, only glucoside and sulfate conjugates of DON, ZON, T-2, and HT-2 have been proven to occur in naturally infected cereals such as maize, wheat, and barley [13]. However, many researchers have carried out studies on the occurrence of free and masked *Fusarium* mycotoxins in cereal-based food and feed commodities (Table 1).

Though toxicological data are scarce since masked mycotoxins represent an emerging condition, studies highlight the potential threat to consumer safety from these substances. However, the possible hydrolysis of masked mycotoxins back to their parent toxin during food/feed processing or during mammalian digestion raises concerns. To ensure the safety of agricultural products (food safety), there is a need for the identification and determination of mycotoxins and their masked forms in order to assess possible effects on consumers. Furthermore, since there are very few investigations on the impact of climatic differences on mycotoxins variation in the different agriculture regions of South Africa, a comprehensive study was imperative.

## 2. Results and Discussion

### 2.1. General Mycotoxin Occurrence Data

Mycotoxins produced by *Fusarium* species are found on grains cultivated on every continent. The susceptibility of maize to *Fusarium* fungi and mycotoxins contamination is well documented [51,52,53]. All the maize samples analyzed in this study were contaminated with an average of 5 to 24 out of 42 *Fusarium* mycotoxins, including 0 to 3 masked forms at the same time. The summary data in Table 2 highlights the relevance of fumonisin B1, B2, B3, B4, and A1 in the samples with 98%, 91%, 80%, 82%, and 54% of 123 samples contaminated with maximum contamination levels of 8908, 3383, 990, 1014, and 51.5 µg/kg respectively. Fumonisin B1 was the most common mycotoxin in the maize samples. Fumonisins commonly occur in maize while in the field, predominantly when they are cultivated in warmer regions [54].

Contamination of maize by fumonisins was considered an important risk factor in human oesophageal cancer in the former Transkei region of South Africa and Santa Catarina State, Brazil [55,56]. Fumonisin B1 has also been implicated in the development of neural tube defects in babies of mothers consuming fumonisin-contaminated maize, especially in certain regions of South Africa, China, and Italy [57,58].

Deoxynivalenol occurred in 50% of the samples with a mean concentration of 152 µg/kg (max 1380 µg/kg). Deoxynivalenol DON causes different unspecific symptoms, for example vomiting and diarrhea, also causes loss of weight and feed refusal in livestock, and hence is known as vomitoxin [59]. The presence of DON in maize has often been associated with samples originating from temperate regions such as northern Europe and North America [60]. However, reports emerging from tropical countries, specifically from South Africa, continue to reveal the occurrence of DON in maize and maize products [61]. It has also been reported at a lower concentration than in this study on maize from Burkina Faso at a mean of 31.4 μg/kg, Mozambique at a range of 116–124 μg/ kg [62] and in Nigerian maize at a range of 11–479 μg/kg [63] and at a mean of 225 μg/kg [64].

Furthermore, according to Ediage et al. [65], DON was detected in the urine of toddlers (1.5−5 years) from Cameroon, which also affirms its presence in food commodities originating from sub-Saharan Africa. However, although half of the analyzed samples contained DON, the maximum level at 1380 µg/kg did not exceed the maximum allowable levels for DON in unprocessed maize as was set by South African Regulation in 2016 at 2000 μg/kg.

Forty-one samples were contaminated with zearalenone at a mean concentration of 13.6 µg/kg. The occurrence of ZON in agricultural commodities has not been thoroughly investigated in sub-Saharan Africa. It was first reported on South African maize and subsequently recovered from maize and other commodities elsewhere on the continent [66]. The maize samples analyzed in this study showed a 33% occurrence rate for ZON, with none of the samples exceeding the maximum level of 350 μg/kg for unprocessed maize products according to European commission [67]. Similarly, it was also reported by Boutigny et al. [68] at 33% occurrence in naturally infected field-grown maize samples. Meyer et al. [69] also reported that less than 10% of the South African commercial maize samples were contaminated with ZON. The detected maximum concentration from the samples was 145.6 μg/kg (Table 2). Compared to other reports on the occurrence of ZON in maize samples from other parts of Africa, the levels detected in this study were lower than levels earlier reported by Adejumo et al. [34] in maize samples from Nigeria. Zearalenone was also reported in maize samples from Cameroon with a mean concentration of 68 μg/kg and a maximum concentration of 309 μg/kg [70]. However, the relatively low level of ZON observed in this study somewhat supports the notion that ZON is perhaps a persistent yet minor contaminant of foods/feeds in South Africa [66,69,71] but its significance is in its oestrogenic potential to mammals.

Occurrence of HT-2 and T-2 in the samples was at a very low level at 0.8% (1 sample) at maximum concentration of 40.2 and 148.0 µg/kg, respectively. The EU permissible levels of mycotoxins by the European Commission have recommended the limit of 15–1000 µg/kg for the sum of HT-2 and T-2 toxins in various matrices [72]. The detected maximum concentration of HT-2 and T-2 from the samples did not exceed the recommended limit. Meanwhile, HT-2 toxin and T-2 are two related compounds that may be synthesized by several *Fusarium* species. Their presence in cereal grain has been well documented, with several reports originating from the cold climate of northern Europe [73,74]. Nevertheless, few reports from Africa indicated; 1–8% incidence rates for HT-2 in Nigerian cereals [64] and 25% rate in Tanzanian maize for HT-2, with range of 15–25 ppb [75]. Also, South African Grain Laboratory (SAGL) reported detection of HT-2 and T-2 only in one maize sample, with the levels 72 and 232 μg/kg, respectively [76].

Nivalenol occurred only in 11% of the samples at a contamination range of 7.7–35.7 μg/kg. Occurrence of NIV in maize has previously been reported in Nigerian samples, although at a higher incidence rate (54%) [63] and higher contamination range of 163–271 μg/kg [64]. Nivalenol is known to be immunosuppressive and also as a protein inhibitor.

Of the masked mycotoxins, DON-3-glucoside occurred at highest incidence rate of 53% than the other masked forms detected in this study. Among emerging toxins, moniliformin, fusarinolic acid, and beauvericin showed high occurrence being found in 98%, 98%, and 83% of samples, respectively. High incidences of these toxins in maize which serve as a staple food in South Africa is an important cause for concern. From available literature, little or no appreciable study has been done on the occurrence of these mycotoxins in food and food products in South Africa and neglecting them increases the risk of exposure to humans and animals. The occurrences of these emerging mycotoxins produced by *Fusarium* specie, have been reported in food crops which represents an important problem in some parts of the world [77,78]. 

Furthermore, the high incidence of these emerging toxins should not be taken for granted, since moniliformin is known to be cytotoxic to many mammalian systems [10]. Beauvericin is also known to be genotoxic to human lymphocytes [79]. Other detected metabolites included emerging mycotoxins and other less well-known Fusarium metabolites such as enniatins (B, B1, and B2), monoacetoxyscirpenol, diacetoxyscirpenol, equisetin, epi-equisetin, aurofusarin, apicidin, neosolaniol, culmorin, 5- & 15-hydroxyculmorin, bikerverin, butenolide, chlamydospordiol, chlamydosporol, chrysogin, fusaric acid, fusapyron, deoxyfusapyron, 7-hydroxypestalotin, acuminatum (B and C) fusarinolic acid, and fusarin C. The co-occurrence of these emerging mycotoxins with other major mycotoxins and many other *Fusarium* metabolites of unknown toxicity is a source of concern. Beauvericin, like enniatins, is a cyclodepsipeptide that has antibiotic, insecticidal, and cytotoxic properties presumably related to their ionophoric properties [80]. 

### 2.2. Distribution of Fusarium (Free and Masked) Mycotoxins across the Agriculture Regions of South Africa

Mycotoxins are among the food-borne risks that are dependent upon climatic conditions. Indeed, the ability of fungi to produce mycotoxins is largely influenced by temperature, relative humidity, insect attack, and stress conditions of the plants [81]. The main effect due to agriculture regions (AR) on the distribution of *Fusarium* free and masked mycotoxins in the maize samples is presented in Table 2.

All the 42 *Fusarium* toxins and metabolites investigated in the maize samples across the agricultural regions were detected and quantified except for the emerging toxin, enniantin B2, which was only detected in 2% of the samples from the western region but was not quantified because the values were below the limit of quantification (<LOQ). Of the major mycotoxins, HT-2 was not detected at all in the eastern region but was quantified only in 2% of the maize from the western region at 40.2 μg/kg concentration. Detection of HT-2 in the warm western region and not the cold eastern region as would be expected is contrary as it was reported that HT-2 was previously considered a problem in colder European climates [13]. Of the fumonisin Bs, FB1 occurred at the most frequency rate than FB2, FB3, and FB4. Fumonisin B1 was the most contaminant mycotoxin, occurring at a mean concentration of 752 ± 1469 μg/kg from the warm western region and of 440 ± 514 μg/kg in the cold eastern region with only 3% (2 samples) not contaminated. A similar trend was also observed when the sum of the fumonisins was considered, for instance, the highest fumonisin (FB1 + FB2 + FB3) concentrations were detected in warm western region and this is consistent with Munkvold [82] and De La Campa et al. [83] who reported optimum temperatures of 30 and 32 °C for fumonisin production. This observation could be correlated to the high mycotoxins production potentials of *Fusarium* fungi in warmer climates [84]. In general, fumonisin B1 was detected in 98% of all the samples analyzed, irrespective of the maize type and AR, ranging from 12.6 to 8908 μg/kg with the mean concentration of 596 μg/kg (Table 2).

Fumonisin B^2^ was the second most contaminant mycotoxin, across the ARs and generally, fumonisin A1 was quantified in 32% of all the maize samples analyzed and in 22% of the samples, it was detected but below the limit of quantification (<LOQ).

Deoxynivalenol was more prevalent in the eastern region than in the western region, having 60% and 40% of the samples all detected and quantified respectively (Table 2). This was in line with what was reported by Chilaka et al. [64] who observed the highest incidence rate of DON in maize samples from the DS zone in Nigeria, (an agricultural region) characterized by a lower temperature and higher average annual. Zearalenone, nivalenol, HT-2, and T-2 were also among the major *Fusarium* mycotoxins, detected and quantified in the two regions. Nivalenol, HT-2, and T-2 were detected in higher concentration in the WR while ZON occurred at lower concentration in the same region, occurring higher in the ER. This observation could be because ZON is known to be produced in somewhat cool environments compared to other mycotoxins [9,22]. The highly toxic mycotoxin, T-2 toxin, has until recently not been recorded in South Africa. T-2 toxin is most commonly produced by *F.* sporotrichioides, a fungus well-adapted to survive in colder countries [85]. Some T-2-producing *Fusarium* species have occasionally been isolated from wheat with *Fusarium* head blight (FHB) and maize with *Fusarium* ear rot (FER) symptoms in South Africa [86]. The presence of T-2 toxin in local maize grain, was reported by the SAGL, [76] and it has been shown to have a relationship with *F*. *verticillioides* and *F. graminearum* [87,88]. 

Masked mycotoxins detected in the samples include DON-3-glucoside, zearalenone-sulphate, hydrolysed fumonisin B1, and HT-2-glucoside. Deoxynivalenol-3-glucoside (DON-3-G) and HT-2-glucoside had the highest and least occurrence respectively across the two regions. Although there were no significant differences in their distribution across the agriculture regions, but to our knowledge, there seem to be no available data on the occurrence of some of these masked mycotoxins in South Africa. Hence, this is the first report of zearalenone-sulfate and HT-2-glucoside in South African maize. Although toxicological data are still limited, the occurrence of masked mycotoxins is expected to add substantially to the overall mycotoxins load and toxicity. This invariably will increase the toxic health effects by these masked mycotoxins, which may be either direct or indirect through hydrolysis, or released from the matrix during digestion into the free mycotoxins [89]. 

Moreover, monoacetoxyscirpenol, diacetoxyscirpenol, neosolaniol, enniatins (B and B1) were not detected in maize samples from the eastern regions but had low incidences of 2%, 3%, 2%, 3%, and 3% respectively in the western region. This could be explained by the agroclimatic differences in the agriculture regions that favored the accumulation of these emerging mycotoxins in the warmer and drier climate of the western maize regions.

### 2.3. Agricultural Region and Maize Type on Fusarium Mycotoxin Distribution and Accumulation on Maize

Mycotoxin occurrence and distribution is influenced by different factors such as crop species, climatic, and environmental conditions of a given region. The interaction between agricultural region and maize type on the concentration of *Fusarium* mycotoxins on maize samples is presented in Table 3.

From the statistical analyses, the concentration of the major mycotoxins; fumonisin Bs (B1, B2, B3, B4) and fumonisin A1 in white maize samples collected from the western region (WR) was significantly higher (*p* < 0.05) than that of yellow maize from the same region, as well as on white and yellow maize from the eastern region (ER). The mean values were 1023 ± 1698, 377 ± 645, 146 ± 197, 140 ± 207, and 10.7 ± 12 µg/kg respectively. The fact that the white maize samples of the western region (WR) had significantly higher mean levels of fumonisin can be explained partly by high mycotoxins production potentials of *Fusarium* fungi in warmer climates as reported by [84]. Then, Munkvold [82] and De La Campa et al. [83] also reported optimum temperatures of 30 and 32 °C for fumonisin production, which is what is obtained in the western region. Furthermore, differences in plant genotype cannot be ruled out as it has been reported that white maize is a better substrate for fumonisin production than yellow maize [90]. 

Deoxynivalenol concentration in yellow maize samples from the western region was significantly higher (*p* < 0.05) than that of the white maize samples from the same region and on the white and yellow maize samples from the eastern region, with the mean value being 367 ± 503 µg/kg (Table 3). Occurrence of DON at significantly higher concentration in the western region could be explained by high mycotoxins production potentials of *Fusarium* fungi in warmer climates as reported by [84]. The higher DON concentration in yellow maize than the white maize could be due to variation in localization of pigments in yellow maize kernel, which might have a prominent role in the actual degree of resistance to *Fusarium* infection/mycotoxin accumulation [91]. 

Zearalenone and nivalenol concentrations showed no significant difference (*p* > 0.05) amongst white and yellow maize samples collected from the western region, as well as white and yellow maize from the eastern region. In addition, white maize from the eastern region has the highest mean concentration of 21.0 ± 47 µg/kg. This is in line with the finding of Ediage et al. [92], who noted no significant difference in zearalenone accumulation in maize samples from Cameroon, irrespective of geographical location.

The concentration of the masked mycotoxins DON-3-glucoside in yellow maize samples from the western region was significantly higher (*p* < 0.05) than that of the white maize samples from the same region and with the yellow maize samples from the eastern region. A similar trend was also observed with the parent toxin deoxynivalenol in this study. This can also have the same explanation as is the case with DON accumulation.

DON-3-glucoside concentration in white maize samples from the eastern region had no statistically significant difference (*p* > 0.05) with the yellow maize samples from the same region and with white maize samples from the western region. In addition, yellow maize from the western region had the highest concentration with mean value of 25.9 ± 36 µg/kg.

Zearalenone sulfate concentrations had no significant differences (*p* > 0.05) amongst white and yellow maize samples collected from the western region, white and yellow maize from the eastern region. A similar trend was also observed with the parent mycotoxin, zearalenone, in this study.

The concentration of hydrolyzed fumonisin B1 in white maize samples collected from the western region was significantly higher (*p* < 0.05) than that of yellow maize from the eastern region. The same trend was observed with other fumonisin parent mycotoxins. Also, hydrolyzed fumonisin B1 in white maize samples collected from the western region had no significant difference (*p* > 0.05) with that of the yellow maize samples from the same region and also with the white maize samples from the eastern region. HT-2-glucoside occurred only on white maize from the western region at mean contamination level of 31.2 ± 0 µg/kg.

Of the emerging toxins, the concentration of 15-Hydroxyculmorin, moniliformin, aurofusarin, and butenolid on yellow maize from the western region was significantly higher (*p* < 0.05) than that of the white maize samples from the same region, as well as white and yellow maize from the eastern region. The mean values were 410 ± 597, 331 ± 327, 643 ± 1490, 118 ± 97 µg/kg respectively. Bikaverin, epiquisetin and equisetin concentrations in yellow maize samples from the eastern region was significantly higher (*p* < 0.05) than that of the white maize samples from the same region and with white and yellow maize samples from the western region. The concentrations of deoxyfusapyron and fusaric acid in white maize samples collected from the western region (WR) is significantly higher (*p* < 0.05) than that of yellow maize from the same region, white and yellow maize from the eastern region. Fusarinolic acid and chrysogin concentrations had no significant difference (*p* > 0.05) amongst white and yellow maize samples collected from the western region, white and yellow maize from the eastern region. Then, beauvericin and acuminatum B concentrations in yellow maize samples collected from the western region is significantly higher (*p* < 0.05) than that of yellow and white maize from the eastern region and had no significant difference (*p* > 0.05) with white maize samples from the western region.

## 3. Conclusions and Recommendations

The analysis showed that maize types were contaminated with a mixture of both free, masked, and emerging mycotoxins across the maize producing regions of South Africa. All the maize samples analyzed had an average of 5 up to 24 out of 42 mycotoxins, including 1 to 3 masked forms at the same time. This study has also shown that there is higher risk of *Fusarium* mycotoxins exposure, especially fumonisin Bs, with consumption of maize grown in the western than with the eastern agriculture regions of South Africa. White maize samples from the western region (WR) had significantly higher mean levels of fumonisins. It also showed that there is no significant difference in the occurrence of the masked toxins across the agriculture regions. Although toxicological data are still limited, the occurrence or presence of masked mycotoxins will add substantially to the overall mycotoxin load and toxicity. However, studies highlight the potential threat to consumer safety from these substances.

## 4. Materials and Methods

### 4.1. Chemicals and Standards

The reagents and chemicals used were of analytical grade (LC gradient grade and MS grade), and were obtained from Merck and Co, Sigma or Microsep, Pretoria, South Africa, except others from Sigma-Aldrich (Vienna, Austria), Merck (Darmstadt, Germany) and VWR (Leuven, Belgium). Mycotoxin standards were obtained from various research groups or purchased from various commercial sources (Romer Labs^®^Inc. (Tulln, Austria), AnalytiCon Discovery (Potsdam, Germany), Bio Australis (Smithfield, Australia). Water was purified successfully by reverse osmosis using Purite™ water purification technology Suez, UK from LASEC, South Africa.

### 4.2. Sampling Site Description

The agriculture regions are the eastern region (ER), which is situated in the higher rainfall and cooler areas and the western region (WR), which is located in the drier and warmer areas. The ER consists of Gauteng province and the eastern Free State where the mean maximum temperatures ranged from 24 to 27 °C while the WR consists of the northwest province and western Free State, where the average temperature ranged from 29 to 32 °C. The geography and climatic conditions of the regions have previously been documented [68]. 

### 4.3. Sampling and Sample Preparation

A total of 123 maize samples harvested during the 2015/2016 season were collected from randomly selected silo sites in the northwest, Gauteng, and Free state province in the two agriculture regions. From the silos located at the western region (WR), 65 composite samples were collected, comprising of 44 white maize and 21 yellow maize. From the eastern region (ER) silo locations, 58 composite samples were collected, comprising of 30 white maize and 28 yellow maize. For each maize sample, at least 10 incremental samples of 100 g each were taken and combined, making approximately 1 kg according to EC 401/2006, in sterile zip lock polythene bags, well labelled and transported to the laboratory. The samples were thoroughly mixed and milled using a sterile high-speed laboratory blender (IKA M20, Merck, Sarstedt, Nümbrecht, Germany) and packaged in sealed sterile plastic bags to avoid contamination. A cleaning and decontamination routine of the equipment was performed using 70% methanol, after each milling practice. Samples were stored prior to analysis at 4 °C in the freezer. The milled sample material was vigorously homogenized with a spatula before weighing.

### 4.4. Fusarium Mycotoxins Analysis

#### 4.4.1. Sample Preparation and Cleanup for LC-MS/MS Multi-mycotoxin Analyses

This was done according to Sulyok et al. [93], sample preparation was rather simple and eludes any clean-up. Briefly, 5 g representative amount of ground maize kernels was weighed into a 50 mL polypropylene tube (Sarstedt, Nümbrecht, Germany) and extracted for 90 min at 180 rpm on a GFL 3017 rotary shaker (GFL, Burgwedel, Germany) with 20 mL of extraction solvent, (acetonitrile/water/acetic acid 79:20:1, v/v/v). Subsequently, the extracts were centrifuged for 2 min at 3000 rpm (radius 15 cm) on a GS-6 centrifuge (Beckman Coulter Inc., Fullerton, CA, USA). The raw extracts were transferred into glass vials using Pasteur pipettes, and 350 μL aliquots were diluted in the same volume (1/1) with dilution solvent, (acetonitrile/water/acetic acid 20:79:1, v/v/v) to adjust the solvent strength. After appropriate mixing, 50 μL of the diluted extract was analyzed by LC-MS/MS without further pre-treatment.

#### 4.4.2. LC-MS/MS Multi-mycotoxin Measurement Parameters

Analyses for the *Fusarium* toxins was performed by liquid chromatography–tandem mass spectrometry (LC-MS/MS) multi-mycotoxin method, at the Centre for Analytical Chemistry, Department of Agro biotechnology (IFA-Tulln), University of Natural Resources and Life Sciences, Vienna, Austria. The analysis was performed according to the methods described by Sulyok et al. [93] and Malachová et al. [94] with slight modifications.

Briefly, a QTrap 4000 LC-MS/MS System (Applied Biosystems, Foster City, CA) was equipped with a turbo ion spray electrospray ionization (ESI) source and an 1100 Series HPLC System (Agilent, Waldbronn, Germany). Chromatographic separation of the analytes was performed at 25 °C on a Gemini^®^ C_18_ column, 150 × 4.6 mm i.d., 5 μm particle size, equipped with a C_18_ 4 × 3 mm i.d. security guard cartridge (Phenomenex, Torrance, CA, US), using (eluent A) methanol/water/acetic acid 10:89:1 (v/v/v) or (eluent B), methanol/water/acetic acid 97:2:1. Both eluents contained 5 mM ammonium acetate. After an initial time of 2 min at 100% A, the proportion of B was increased linearly to 100% within 12 min, followed by a hold-time of 3 min at 100% B and 4 min column re-equilibration at 100% A. The injection volume of 50 µL and flow rate of 1 mL min^−1^ was used.

The ESI-MS/MS source temperature operated at 550 °C, in the multiple reaction monitoring (MRM) mode, both in positive and negative polarities in two separate chromatographic runs per sample by scanning two fragmentation reactions per analyte. Further MS parameters were as follows: curtain gas 10 psi (69 kPa of max. 99.5% nitrogen); ion source gas 1 (sheath gas) 50 psi (345 kPa of nitrogen); ion source gas 2 (drying gas) 50 psi (345 kPa of nitrogen); ion spray voltage −4000 V and +4000 V respectively, collision-activated dissociation gas (nitrogen) high.

#### 4.4.3. Method Validation

LC-MS/MS multi-mycotoxins method was validated in terms of linearity, apparent recovery (AP), limit of detection (LOD), limit of quantification (LOQ), (Table 1), using blank matrices of maize, (401/2006/EC, 2006). Apparent recoveries of the analytes were calculated by spiking five different samples that were not contaminated with mycotoxins with a multi-analyte standard. The spiked samples (0.25 g) each were left overnight in the dark at room temperature for evaporation of the solvent to establish equilibrium between the analytes and the sample was then extracted with 1 mL of extraction solvent as described above. The corresponding peak areas of the spiked samples were used for estimation of the apparent recovery by comparison with a standard of the same concentration prepared by dilution in pure solvent:

$$\mathrm{RA}\%=100\times \frac{\mathrm{Peak}\;\mathrm{area}\;\mathrm{spiked}\;\mathrm{samples}}{\mathrm{Peak}\;\mathrm{area}\;\mathrm{liquid}\;\mathrm{standards}}$$

Limits of detection (LOD) and limit of quantification (LOQ) were calculated from the signal to noise ratios (S/N) of the respective multiple reaction monitoring (MRM) chromatograms deriving from analysis of the spiked samples: LOD = 3 × S/N and LOD = 10 × S/N, respectively.

#### 4.4.4. Data Analysis

The data collected were subjected to analyses of variance (ANOVA), as outlined for factorial arrangement in a completely randomized design (CRD) using GenStat Release 10.3DE [95] statistical software. The means were compared using Fisher’s least significant difference (F-LSD) at 5% probability level. The data were not log-transformed, only the geometric means were taken. Microsoft Office Excel software version 2016 was used to determine the frequencies, range, and percentages.

## Figures and Tables

**Table 1:** Occurrence data of glucoside and sulfate conjugates (masked mycotoxins) and their respective free forms identified in cereal grains and food/feed products.

Mycotoxin	Food/Feed Commodity	% Positive Samples	Range (µg/kg)	References
**ZON**	Maize	83	59–1071	[14]
	Maize	-	<LOQ-15,700	[15]
	Maize	-	56	[16]
	Maize	37%	<50–196	[17]
	Maize	90	0–135	[18]
	Maize	-	14	[19]
	Maize	-	10.2	[20]
	Maize	92.5	247.8	[21]
	Maize silage(feed)	79.4	20-11	[22]
	Barley	5.9	<LOQ-17	[23]
	Barley	67	2–31	[24]
	Beans	90	185.2	[21]
	Wheat	46.7	<LOQ-234	[23]
	Wheat	83.3	12–109	[14]
	Wheat	47.5	1–100	[24]
	Peanut	57	70	[25]
	Oat	41.9	<LOQ-675	[26]
	Oat	66.7	13–85	[14]
	Oat	100	5–15	[24]
	Cocoa seeds	23.5	24.2–83.6	[27]
	Bread	83.3	19–53	[14]
	Breakfast cereals	-	4	[28]
	Corn flakes	83.3	34–90	[14]
	Malt	56	102–2213	[29]
	Traditional brewed beer	45	2.6–426	[30]
**ZON-14G**	Maize	17	274	[14]
	Maize	-	<LOQ-9750 (total)	[14]
	Barley	17.6	<LOQ-9.6	[23]
	Wheat	6.7	<LOQ-0.6	[23]
	Wheat	0	-	[14]
	Oats	3.2	<LOQ	[23]
	Oats	0	-	[14]
	Bread	33.3	20–20	[14]
	Corn flakes	0	-	[14]
**ZON-16G**	Barley	23.5	<LOQ	[23]
	Wheat	6.7	<LOQ-2.8	[23]
	Oat	58.1	<LOQ-7.9	[23]
**ZON-14S**	Maize	17	51	[14]
	Maize	-	<LOQ-9750 (total)	[15]
	Maize silage(feed)	42.3	2–4318	[22]
	Barley	8.8	<LOQ-26.1	[23]
	Wheat	33.3	11	[14]
	Wheat	40.0	<LOQ-22.5	[23]
	Oats	16.7	12	[14]
	Oats	29.0	<LOQ-220	[23]
	Bread	16.7	24	[14]
	Corn flakes	0	-	[14]
**DON**	Maize	100	255–5245	[14]
	Maize	100	90–680	[31]
	Maize	100	32–2246	[32]
	Maize	90	74–1382	[33]
	Maize	22	9.6–745.1	[34]
	Maize	100	1,469	[20]
	Maize	100	0.3–4374	[32]
	Maize	-	380	[16]
	Maize silage (feed)	71.8	1.5–13,488	[22]
	Barley	82.4	LOQ-1180	[23]
	Barley	100	<60	[32]
	Barley	83	54–1602	[24]
	Whole wheat	-	8000	[28]
	Wheat	83	11–1265	[13]
	Wheat	97.6	LOQ-5510	[23]
	Wheat	66.7	16–150	[14]
	Wheat	100	540–5080	[31]
	Wheat	100	46–2683	[32]
	Wheat	46.5	25–2975	[24]
	Wheat	46.1	LOQ-297	[35]
	Wheat	75	LOQ-10,130	[36]
	Wheat	-	40–490	[37]
	Wheat	68	up to 302	[38]
	Rice	23.8	107.9	[39]
	Durum wheat	100	1750	[36]
	Rye	100	<50	[32]
	Oat	100	2690	[23]
	Oat	16.7	46	[14]
	Oat	81.8	62–2216	[32]
	Oat	100	67–149	[24]
	Bread	66.7	20–102	[14]
	Corn flakes	16.7	207	[14]
	Breakfast cereals	28.6	28.6	[40]
	Snacks	61.8	61.8	[40]
	Flours	72.3	72.3	[40]
	Wheat flour	97.2	1.3–825.9	[41]
	Wheat flour	100	78.9–325.8	[35]
	Swine feed	93.8	50–931	[33]
	Poultry feed	93.3	157–1231	[33]
	Feed	99	124–2352	[42]
	Lager beer	100	1.6–6.4	[43]
	Beer	90	LOQ-35.9	[44]
**DON-3G**	Maize	100	36–1003	[14]
	Maize	67	<LOQ-70	[31]
	Maize	100	<35	[32]
	Maize	80	14–121	[33]
	Maize	-	<LOQ-1100	[15]
	Maize silage(feed)	63.0	1.0–3159	[22]
	Barley	73.5	<LOQ-1300	[23]
	Barley	0	-	[32]
	Barley	29	43–277	[24]
	Wheat	83.3	<LOQ-922	[23]
	Wheat	16.7	18	[14]
	Wheat	100	59–200	[31]
	Wheat	83.3	43–737	[32]
	Wheat	27.3	40-356	[24]
	Wheat	27	16-138	[45]
	Wheat	31	<LOQ	[35]
	Wheat	75	100-1230	[36]
	Durum wheat	94	LOQ-850	[46]
	Rye	0	-	[32]
	Oat	87.1	<LOQ-6600	[23]
	Oat	100	28–97	[14]
	Oat	45.5	162–287	[32]
	Oat	0	-	[24]
	Bread	83.3	26–29	[14]
	Corn flakes	50.0	24–28	[14]
	Breakfast cereals	85.7	19–66	[40]
	Snacks	82.4	11–94	[40]
	Flours	68.2	5–72	[40]
	Wheat flour	33.4	0.2–15.7	[41]
	Swine feed	93.8	6–80	[33]
	Poultry feed	93.3	30–107	[33]
	Beer	95	LOQ-27.5	[44]
**NIV**	Barley	73.5	<LOQ-302	[23]
	Wheat	43.3	<LOQ-74.0	[23]
	Oat	71.0	<LOQ-4940	[23]
	Rice	-	97	[47]
	Wheat flour	100	LOQ-140.6	[35]
**NIV-3G**	Barley	61.8	<LOQ- 65.3	[23]
	Wheat	43.3	<LOQ-33.6	[23]
	Oat	16.1	<LOQ-58.3	[23]
**HT-2**	Barley	35.3	<LOQ-39.5	[23]
	Barley	100	23–233	[48]
	Barley	100	3–213	[49]
	Wheat	100	19–96	[48]
	Wheat	63.3	<LOQ-39.5	[23]
	Wheat	77.8	26–85	[50]
	Oat	100	11–187	[48]
	Oat	74.2	<LOQ-1830	[23]
	Oat	88.9	21–851	[50]
**HT-2G**	Barley	52.9	<LOQ-38.5	[23]
	Barley	94.4	0.6–162.8	[49]
	Wheat	53.3	<LOQ-15.9	[23]
	Oat	16.1	<LOQ-300	[23]
**T-2**	Barley	100	41–160	[48]
	Barley	100	1–154	[49]
	Wheat	100	17–76	[48]
	Wheat	55.6	11–23	[50]
	Oat	100	31–165	[48]
	Oat	88.9	10–377	[50]
	Maize	36	7.5–29	[17]
**T-2G**	Barley	50	0.1–14.5	[50]

**Table 2:** Summary statistics of occurrence of investigated 42 *Fusarium* free mycotoxins, masked and emerging mycotoxin metabolites in 123 maize samples on LC-MS/MS.

	Concentration (µg/kg)			Number of Positive Samples Across ARs ^a^		N ^b^ (123)	P ^c^ %	LOQ ^d^	LOD ^e^	Rec ^f^ (%)
Metabolite Group	Minimum	Maximum	Mean	ER (n = 58)	WR (n = 65)					
**Free *Fusarium* Mycotoxins**										
Fumonisin B_1_	12.6	8908	596	58	63	121	98	8	2.4	75.0
Fumonisin B_2_	7.9	3383	221	55	57	112	91	7	2.1	79.0
Fumonisin B_3_	<LOQ	990	85.6	44	54	98	80	7	2.1	75.0
Fumonisin B_4_	<LOQ	1014	81.6	45	56	101	82	7	2.1	75.0
Fumonisin A_1_	<LOQ	51.5	6.1	28	38	66	54	2.1	0.6	75.0
**Fumonisin Total**	**20.5**	**14,347**	**990**	**n/a**	**n/a**	**n/a**	**n/a**	**n/a**	**n/a**	**n/a**
Zearalenone	<LOQ	146	13.6	19	22	41	33	0.6	0.2	54.0
HT-2 toxin	40.2	40.2	40.2	0	1	1	0.8	6.4	1.9	96.8
T-2 toxin	148.0	148	148	0	1	1	0.8	2.4	0.8	102.0
Deoxynivalenol	8.2	1380	152	35	26	61	50	-	1.2	85.0
Nivalenol	7.7	35.7	14.2	4	10	14	11	3.8	1.1	80.0
**Masked *Fusarium* Mycotoxins**										
DON-3Glucoside	2.43	112	15.0	31	34	65	53	-	0.8	92.0
ZON-sulphate	11	146	14.0	9	13	22	18	n.a	-	75.0
Hydrolysed FB_1_	0.7	28.0	3.9	12	19	31	25	0.7	0.2	100.0
HT-2 Glucoside	31.2	31.2	31.2	0	1	1	0.8	5.7	1.7	71.9
**Emerging Mycotoxins and Other Less Well-Known *Fusarium* Metabolites**										
Monoacetoxyscirpenol	20.9	20.9	20.9	0	1	1	0.8	5.2	1.6	89.0
Diacetoxyscirpenol	4.4	5.0	4.7	0	2	2	1.7	0.5	0.2	81.8
Neosolaniol	4.5	4.5	4.5	0	1	1	0.8	4.6	1.4	83.3
Culmorin	13.3	465	90.0	7	11	18	15	5.5	1.6	76.0
15Hydroxyculmorin	<LOQ	2022	181	24	25	49	40	20.8	6.2	55.7
5Hydroxyculmorin	<LOQ	578	167	12	6	18	15	50	15	102.0
Moniliformin	<LOQ	1130	219	57	63	120	98	5	1.5	100.0
Beauvericin	<LOQ	142	7.2	56	51	107	87	0.03	0.01	100.0
Enniatin B	0.1	4.9	2.5	0	2	2	1.7	0.04	0.01	81.0
Enniatin B_1_	0.13	3.0	1.6	0	2	2	1.7	0.11	0.03	100.0
Enniatin B_2_	<LOQ	<LOQ	<LOQ	0	1	1	0.8	0.57	0.2	91.2
Aurofusarin	<LOQ	5470	296	46	43	89	72	3.7	1.1	79.8
Bikaverin	<LOQ	651	72.0	38	44	82	67	55	16	79.6
Butenolid	<LOQ	214	48.9	22	13	35	28	10.8	3.2	61.0
Epiequisetin	<LOQ	18.9	5.0	7	12	19	15	2	0.6	70.3
Equisetin	<LOQ	129	19.4	14	16	30	24	2.3	0.7	79.9
Apicidin	2.9	15.4	9.1	1	1	2	1.6	0.65	0.2	108.0
Deoxyfusapyron	<LOQ	53.0	11.1	7	16	23	19	2.7	0.8	103.0
Fusapyron	<LOQ	18.0	11.1	14	24	38	31	2.4	0.7	106.0
Fusaric acid	57.9	195	85.2	14	10	24	20	30	10	91.4
Fusarinolic acid	<LOQ	3422	506	57	63	120	98	30	10	89.5
7Hydroxypestalotin	<LOQ	16.5	7.5	8	17	25	20	2.9	0.9	100.0
Acuminatum B	<LOQ	219	33.0	4	8	12	9.8	30	10	105.0
Acuminatum C	26.5	204	98.8	4	3	7	5.7	30	10	62.4
Chlamydospordiol	2.1	5.1	7.3	1	1	2	1.7	0.3	0.1	96.5
Chlamydosporol	27.0	26.9	26.9	0	1	1	0.8	1.1	0.3	94.0
Chrysogin	<LOQ	7.7	4.4	6	42	48	30	1.9	0.6	99.2
Siccanol	34.6	252	64.3	43	48	91	74	-	-	86.9

^a^ Contamination across agricultural regions (ARs), eastern region (ER), western region (WR). ^b^ Total number of contaminated samples (N). ^c^ Percentage of total number of samples contaminated (P). ^d^ LOQ: limit of quantification, <LOQ: less than LOQ. ^e^ LOD: limit of detection [s/n = 3:1] expressed as µg/kg sample. ^f^ Recovery: calculated from spiking experiment of maize samples. n/a: Not applicable.

**Table 3:** Occurrence of *Fusarium* mycotoxins in white and yellow maize from the agriculture regions.

	ER		WR		
Free *Fusarium* Mycotoxins	WM (Mean ± SD)	YM (Mean ± SD)	WM (Mean ± SD)	YM (Mean ± SD)	LSD (<0.05)
	Concentration (µg/kg)				
Fumonisin B_1_	607 ± 579	273 ± 362	1023 ± 1698	482 ± 634	407.26
Fumonisin B_2_	200 ± 189	102 ± 121	377 ± 645	203 ± 248	156.06
Fumonisin B_3_	80.9 ± 60	43.5 ± 35	146 ± 197	7.2 ± 59	4.87
Fumonisin B_4_	71.6 ± 53	39.7 ± 32	140 ± 207	75.1 ± 74	52.61
Fumonisin A_1_	4.2 ± 2	3.7 ± 1	10.7 ± 12	5.8 ± 3	4.37
Zearalenone	21.0 ± 47	9.8 ± 13	4.4 ± 9	19.2 ± 27	17.64
Deoxynivalenol	121 ± 186	51.0 ± 35	71.0 ± 71	367 ± 503	136.80
Nivalenol	10.2 ± 0	15.3 ± 4	15.8 ± 9	15.5 ± 6	6.79
T-2	<LOD	0	148 ± 0	0	-
HT-2	0	0	31.2 ± 0	0	-
**Masked *Fusarium* Mycotoxins**					
DON-3-glucoside	18.6 ± 25	6.7 ± 4	8.7 ± 11	25.9 ± 36	13.73
Zearalenone-Sulphate	2.78 ± 48	1.14 ± 97	7.36 ± 54	9.5 ± 71	22.30
Hydrolysed Fumonisin B_1_	3.8 ± 3	1.8 ± 1	6.5 ± 7	3.6 ± 4	3.13
HT-2-glucoside	0	0	31.2 ± 0	0	-
**Emerging Mycotoxins and Other Less Well-Known *Fusarium* Metabolites**					
Monoacetoxyscirpenol	0	0	20.9 ± 0	0	-
Diacetoxyscirpenol	0	0	4.7 ± 1	0	-
Neosolaniol	0	0	4.8 ± 0	0	-
Culmorin	62.4 ± 25	159 ± 177	22.7 ± 7	116 ± 102	101.47
15Hydroxyculmorin	179 ± 148	79.2 ± 55	55.5 ± 21	410 ± 597	190.36
5Hydroxyculmorin	111 ± 16	58.7 ± 349	167 ± 0	331 ± 0	233.20
Moniliformin	184 ± 178	165 ± 144	195 ± 227	331 ± 327	96.21
Beauvericine	2.3 ± 5	4.4 ± 8	7.8 ± 27	14.2 ± 26	9.24
Enniatin B	0	0	5.0 ± 0	0.1 ± 0	-
Enniatin B_1_	0	0	2.98	0.13	-
Enniatin B_2_	0	0	<LOQ	<LOQ	-
Aurofusarin	130 ± 188	283 ± 577	127 ± 127	643 ± 1490	351.51
Bikaverin	56.1 ± 0	156 ± 0	0	76.3 ± 16	75.81
Butenolid	31.2 ± 14	27.6 ± 11	19.3 ± 9	118 ± 97	42.67
Epiquisetin	1.5 ± 0	11.8 ± 7	4.4 ± 2	2.2 ± 0	4.13
Equisetin	4.2 ± 2	50.7 ± 56	17.8 ± 10	4.7 ± 1	28.94
Apicidin	15.4 ± 0	0	2.9 ± 0	0	-
Deoxyfusapyron	3.3 ± 0	5.0 ± 2	30.1 ± 23	6.1 ± 3	21.74
Fusapyron	3.3 ± 0	5.0 ± 8	30.1 ± 4	6.1 ± 5	21.74
Fusaric Acid	0	109 ± 46	133 ± 26	99.4 ± 22	22.70
Fusarinolic Acid	530 ± 387	493 ± 281	557 ± 590	446 ± 293	161.10
Hydrolysed Fumonisin B_1_	3.8 ± 3	1.8 ± 1	6.5 ± 7	3.6 ± 4	3.13
7Hydroxypestalotin	8.5 ± 4	5.8 ± 1	6.8 ± 3	9.2 ± 4	2.76
Acuminatum B	0	0	27 ± 6	103 ± 82	94.90
Acuminatum C	58.4 ± 25	164 ± 41	129 ± 0	44.8 ± 18	45.80
Chlamydospordiol	2.1 ± 0	-	5.1 ± 0	-	-
Chlamydosporal	-	-	26.9 ± 0	-	-
Chrysogin	5.6 ± 6	3.8 ± 2	3.6 ± 2	4.5 ± 2	2.01
Siccanol	62.0 ± 51.2	42.3 ± 26.2	61.7 ± 59.3	91.0 ± 55.3	24.9

AR—agriculture region, ER—eastern region, WR—western region, Qty—percentage quantified, <LOD—percentage below limit of detection, <LOQ—percentage below limit of quantification, YM—yellow maize, WM—white maize, LSD—least significant difference, SD—standard deviation.
